# Maxillomandibulocardiac reflex in a dog

**DOI:** 10.1186/s13028-018-0421-5

**Published:** 2018-10-29

**Authors:** Luca Bellini, Anna Perazzi, Barbara Carobbi, Ilaria Iacopetti

**Affiliations:** 1 Veterinary Teaching Hospital, University of Padua, Viale dell’Università 16, 35020 Legnaro, PD Italy; 2 Department of Animal Medicine, Production and Health, University of Padua, Viale dell’Università 16, 35020 Legnaro, PD Italy

**Keywords:** Atropine, Dog, Dopamine, Maxillomandibulocardiac reflex, Trigeminocardiac reflex

## Abstract

**Background:**

The trigeminocardiac reflex (TCR) is a brainstem reflex that may be observed in anaesthesia during surgical procedures stimulating the intracranial or peripheral portion of the trigeminal nerve. The peripheral TCR is divided into the oculocardiac reflex and the maxillomandibulocardiac reflex based on the affected sensory branches of the trigeminal nerve. In veterinary medicine the oculocardiac reflex has been described, however the maxillomandibulocardiac reflex has never been reported.

**Case presentation:**

A 5-year-old male Epagneul Breton was presented for surgical management of an upper lip mass. During surgery, a sudden severe bradycardia and a decrease in systemic arterial blood pressure developed. The occurrence of a maxillomandibulocardiac reflex was suspected on the basis of the temporary link between surgical stimulation and haemodynamic changes. Three doses of atropine were given before starting a dopamine infusion due to lack of response. The dopamine infusion normalized heart rhythm and blood pressure. The dog recovered uneventfully and he was discharged 24 h later with a sinus rhythm and no sign of recurrence of arrhythmias.

**Conclusion:**

The TCR is a rare but potentially life-threatening complication of procedures involving the sensory areas innervated by the three branches of the trigeminal nerve and it may cause bradycardia with hypotension. The use of a β1-adrenergic receptor agonist such as dopamine may be indicated in cases of a refractory response to the conventional treatment with atropine.

## Background

The trigeminocardiac reflex (TCR) is a brainstem reflex defined as a sudden decrease of at least 20% in heart rate and/or systemic arterial blood pressure during stimulation of any of the sensory branches of the trigeminal nerve [1]. The TCR is commonly divided into a central type and a peripheral (or ganglion) type based on the location of the trigger points on the trigeminal nerve. In the central TCR the cause of the reflex is an intracranial stimulation of the trigeminal nerve, whereas in the peripheral TCR the stimulation occurs on the nerve portion originating from the trigeminal (Gasserian) ganglion. The peripheral TCR is further divided in two subtypes, oculocardiac and maxillomandibulocardiac, depending on which sensory branch is involved. The afferent pathway of the reflex consists of first order sensory neurons that synapse in the trigeminal sensory nucleus and, via the internucial neurons in the reticular formation, link to the motor nucleus of the vagus that innervates the myocardium [2, 3].

In human anaesthesia, TCR is observed frequently during surgical procedures involving the base of the skull, and cases of TCR are reported during procedures to correct strabismus in children or basal cell carcinoma removal from the skin over the zygomatic arch [4–8]. The incidence of the maxillomandibulocardiac reflex associated with maxillofacial surgeries, nasal endoscopic procedures, and dental extractions or injections is 1.5% in humans [7], however the incidence of this reflex in small animals is unknown.

The only type of TCR reported in veterinary clinical practice is the oculocardiac reflex although the real medical relevance of this condition has been questioned [2, 9]. Two case reports describe an oculocardiac reflex due to a traumatic zygomatic arch fracture, and a choroidal melanoma with orbital extension both compressing the ophthalmic branch of the trigeminal nerve [10, 11]. In both reports, the pre-anaesthetic clinical examination revealed a heart rate lower than expected. Electrocardiogram (ECG) examination showed bradycardia and first-degree atrioventricular (AV) block. In dogs, TCR originated from branches other than the ophthalmic during anaesthesia have not been reported previously.

We describe a case report of a suspect of maxillomandibulocardiac reflex and its management in an Epagneul Breton undergoing surgical excision of a labial mass.

## History

A 5-year-old, male, Epagneul Breton, weighing 15 kg, was admitted for examination and management of a mass located on the internal surface of the upper lip detected by the owner 2 weeks before.

**Fig. 1:**
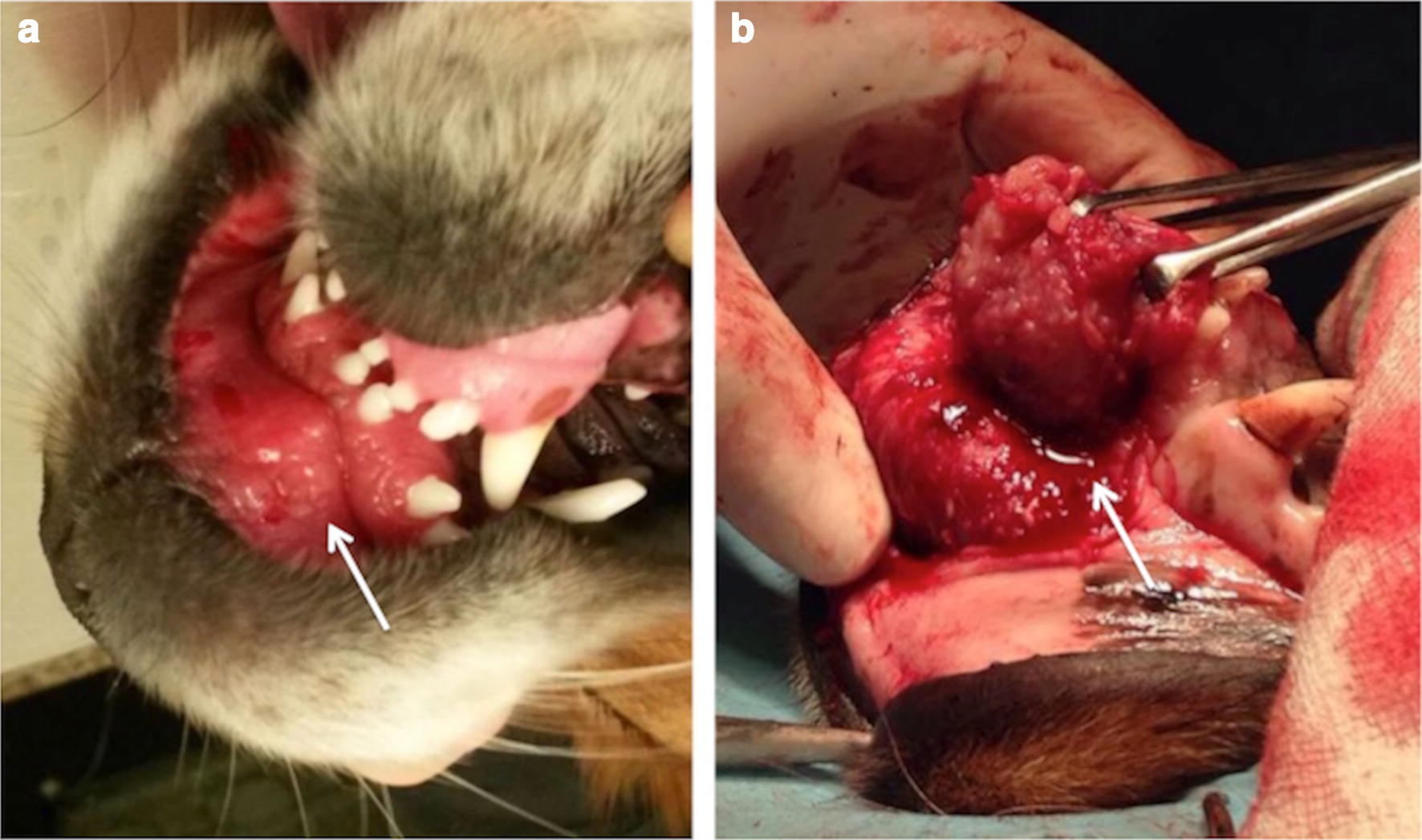
Upper lip mass. **a** Soft tissue mass on the internal surface of the upper lip (white arrow). **b** Intraoperative view of the mass (white arrow) with its subcutaneous attachment at the maxillary incisor bone surface

At physical examination the dog was bright, alert, and responsive. The dog was up-to-date with vaccinations and heartworm (*Dirofilaria immitis*) prophylaxis. No past history of syncope or exercise intolerance were reported by the owner. Rectal temperature was 38.2 °C, heart rate 96 beats/min and respiratory rate 24 breaths/min, mucous membranes were pink and the capillary refill time was 2 s. Thorax auscultation was unremarkable, heart sounds were rhythmic and normal with no audible murmur. A soft tissue mass was palpated on the internal surface of the upper lip, symmetrically developed and straddling the labial frenulum (Fig. 1a). Complete blood cell count and serum biochemistry profile were within reference limits.

Histological examination of a sample of the mass revealed changes consistent with a low grade spindle cell sarcoma. A rostral maxillectomy was recommended, however the owner refused, consequently a computer tomography (CT) scan was scheduled to detect any contraindication to palliative surgery.

A 20 gauge intravenous (IV) catheter was placed in the right cephalic vein and an infusion of lactated Ringer’s solution 5 mL/kg/h was started. Dexmedetomidine (Dexdomitor, Orion Pharma, Milan, Italy) 2 µg/kg and methadone (Semfortan, Eurovet Animal Health, Milan Italy) 0.12 mg/kg were administered intravenously as pre-anaesthetic medication. General anaesthesia was induced with propofol (Vetofol, Norbrook Laboratories Ltd, County Down, Northern Ireland) 2.5 mg/kg and maintained with sevoflurane (Sevoflo, Abbott Laboratories Ltd, Rome, Italy; end-tidal 2.1%) in a mixture of oxygen/air (F_I_’O_2 _= 0.5) administered through a 10 mm ID silicone endotracheal tube.

The CT scan did not reveal any sign of metastasis or maxillary incisor bone lysis, therefore the dog was moved to theatre to perform a palliative surgery.

**Fig. 2:**
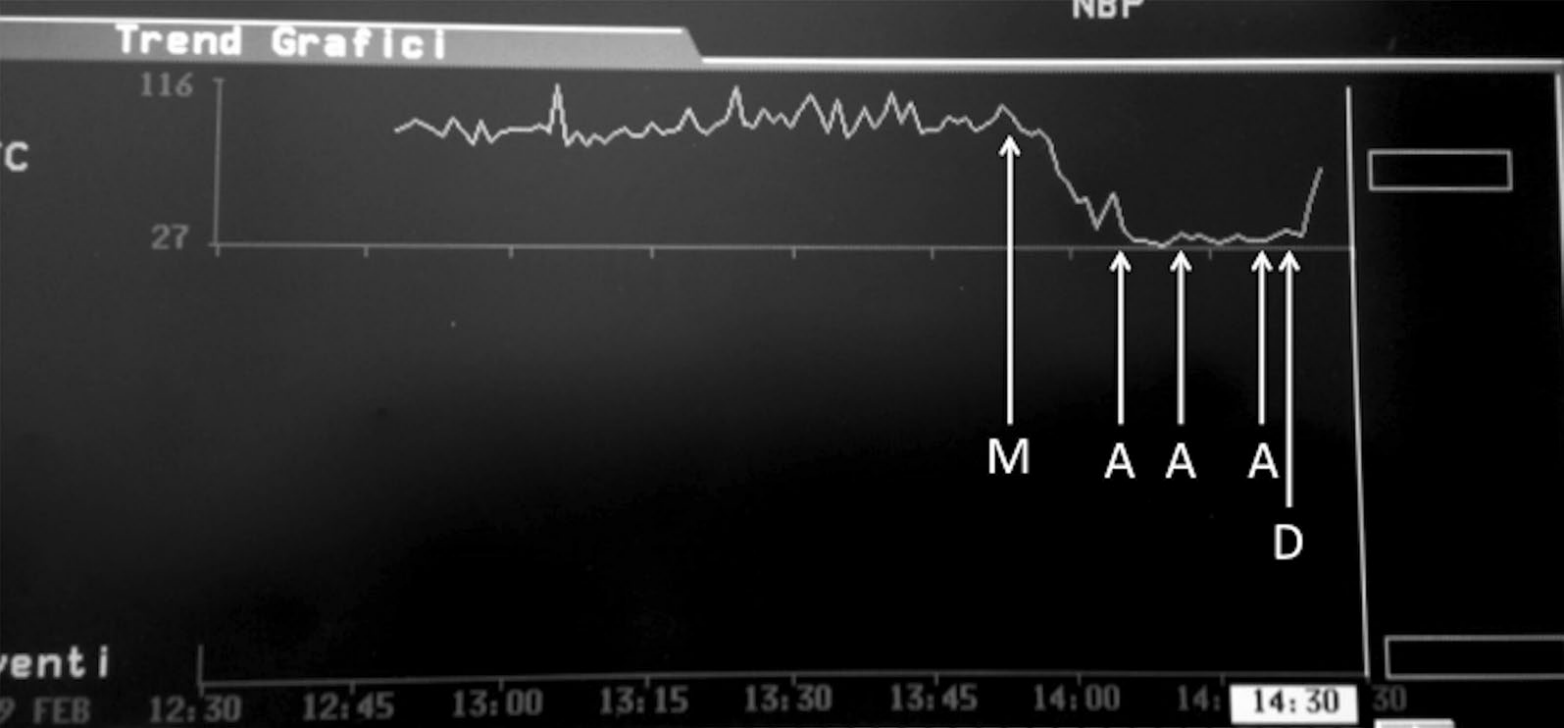
Heart rate trend. M: mass removal. A: atropine (0.02 mg/kg) administration. D: dopamine infusion (10 µg/kg/min)

The dog was positioned in dorsal recumbency and a top-up of methadone 0.1 mg/kg was administered IV. Arterial blood pressure, inspiratory and end-tidal carbon dioxide, ECG, pulse oximetry, heart and respiratory rates, were monitored continuously throughout the procedure. Mechanical ventilation (peak pressure 12 cm H_2_O) was applied to maintain normocapnia (end-tidal carbon dioxide 35–45 mmHg) and an IV infusion of ketamine (Imalgene, Merial Italia, Livorno, Italy) 10 µg/kg/min was started with a loading dose of 0.5 mg/kg. At the beginning of the surgery, a heart rate (HR) between 85 and 95 beats/min was recorded with a sinus rhythm. Anaesthesia was uneventful until 90 min after induction, then the HR progressively decreased from 82 to 29 beats/min within 5 min (Fig. 2). At that time, surgeons were dissecting the connection of the mass to the subcutaneous tissue of the lip at the maxillary incisor bone surface (Fig. 1b). Supraventricular complexes not preceded by P waves were recorded at Lead II ECG, however palpation of the femoral pulse revealed a regular rhythm with frequency corresponding to the QRS complexes on the ECG monitor. Mean arterial blood pressure was 47 mmHg. In order to restore normal rhythm, surgical manipulation was temporarily stopped and IV atropine (Atropina Solfato, ATI Srl, Ozzano dell’Emilia, Italy) 0.02 mg/kg was administered. A second dose of IV atropine 0.02 mg/kg was administered 5 min later because of absence of response and the end-tidal sevoflurane was decreased at 1.6%, the ketamine infusion was stopped. After 10 min of no response, a third dose of IV atropine 0.02 mg/kg was administered, however heart rate did not increase therefore dopamine (Dopamina Cloridrato, Hospira Italia Srl, Naples, Italy) 10 µg/kg/min IV infusion was started 2 min later. Consequently, ventricular discharge rate increased, atrial P waves were back, however they were dissociated from QRS complexes. The mean blood pressure increased to 67 mm Hg. Sevoflurane was maintained at the same end-tidal concentration until the end of the procedure, 15 min later. Then sevoflurane was turned off and the dog was extubated after 10 min. Dopamine was discontinued after 20 min of infusion.

The results of arterial blood gas and electrolyte assays performed at this time were unremarkable. In the postoperative period the ECG was monitored continuously to check for arrhythmias, and showed a sinus rhythm with normal wave morphology and an averaged HR of 78–97 beats/min during the next 24 h. The dog recovered uneventfully from anaesthesia and was discharged into the owner’s care 24 h later.

## Discussion

In human literature the TCR is defined as a sudden onset of bradycardia with or without systemic hypotension, apnoea and gastric hypermotility [6]. TCR is caused by stimulation of one of the sensory branches of the trigeminal nerve; an abrupt drop of HR below 20% or more from the baseline or asystolie may represent the efferent response. In the present case we observed a sudden decrease of the HR (more than 20% from the baseline) during labial surgery.

To confirm the occurrence of such a reflex, it should be observed in a clinical setting, during surgical procedures involving any branches of the trigeminal nerve; moreover the event has to fulfil at least one of two major criteria, plausibility or reversibility, that define a cause–effect relationship [3]. Plausibility requires a temporal link between stimulation or manipulation of the nerve and the haemodynamic reaction. In the present case a sudden onset of bradycardia was observed at the same time as surgical manipulation of the maxillary incisor bone surface. The HR should return to the pre-stimulation condition when the stimulation ceases to satisfy the criterion of reversibility. In the postoperative period, in our patient, HR and rhythm were back to normal.

Moreover, TCR on the basis of the trigger points, is divided into central TCR which is triggered upon stimulation of the intracranial course of the trigeminal nerve, and peripheral TCR which is elicited upon the stimulation of the trigeminal nerve anywhere along its course outside the cranium. Peripheral TCR is further subdivided into oculocardiac reflex and maxillomandibulocardiac reflex based on the branches of the affected trigeminal nerve [1]. In veterinary literature two case reports describe an oculocardiac reflex [10, 11], however a maxillomandibulocardiac reflex has not been described. In the present case the TCR was thought to be related to the stimulation of the maxillary branch. The mass was located on the internal surface of the upper lips, sensory innervation of this area comes from the *rami labiales superiores*, which originates from the infraorbital nerve that separates from the trigeminal maxillary branch and enters the infraorbital channel [12]. Therefore, we assume it was a maxillomandibulocardiac reflex. In most cases, interruption of surgical manipulation eliminates the reflex response, and heart rate return to normal without drug administration. Nevertheless, in the event of persistent bradycardia, the use of atropine is recommended [13]. However this reflex may or may not respond to parasympathicolytic agents because bradycardia is caused by vagal stimulation but also it may result from a reduction in sympathetic outflow [1]. Cases of TCR unresponsive to atropine treatment have been described in two adult human patients undergoing a microvascular decompression of the trigeminal nerve root entry zone and cerebellopontine angle surgery [8, 14]. Moreover the cardiac ventricle is innervated mainly by sympathetic nerve fibres therefore atropine and other antimuscarinic drugs are less effective in restoring ventricular rhythm during atrioventricular conduction abnormalities [15]. To treat this type of abnormalities, it would therefore be recommended to administer a β1-adrenergic receptor agonist which can stimulate heart sympathetic nerve fibres. At the time of surgical manipulation of the maxillary incisor bone surface we recorded a sudden decrease of the HR up to 29 beats/min, which did not normalize despite the temporary interruption of surgical stimulation. We therefore administered 3 doses of atropine (0.02 mg/kg), however HR remained still below 35 beat/min, and, as described in human in unresponsive TCR, a β1-adrenergic receptor agonist was administered (dopamine 10 µg/kg/min) and bradycardia resolved. Atropine-unresponsive bradycardia may occur with other causes, such as hypothermia or hyperkalaemia, but these changes were not present in our case.

Hypotension detected in our case during reflex bradycardia may have resulted from excessive vagal stimulation but also may have been a consequence of a reduction in sympathetic tone. The TCR response may cause a bradycardia with normal systemic arterial blood pressure, although hypotension can develop secondary to a systemic vasodilation mediated by a sympathetic depression [6]. We thought that the observed hypotension was partially due to a decrease in the sympathetic outflow and a vagally mediated negative inotropic effect. In such a situation, in addition to the stimulation of β1-adrenoceptors, the α1-agonist activity of dopamine on arterial vasculature could have helped normalize systemic arterial blood pressure.

Perioperative factors associated with TCR in humans include: light plane of anaesthesia, hypercapnia, hypoxaemia, acidosis, drugs (potent opioids or sevoflurane, β-blockers and calcium channel blockers), and high resting vagal tone [1]. No clinical signs of a light anaesthetic plane or abnormal capnograph were present. Although we did not perform an arterial blood gas analysis during surgery, hypercapnia and hypoxaemia have been excluded as partial pressure of carbon dioxide on expired gases was maintained around 42–48 mmHg and the arterial oxygen saturation was higher than 95% all over the anaesthesia. Additionally the arterial blood gas performed immediately after surgery confirmed a normal pH of 7.370 with a partial pressure of oxygen and carbon dioxide of 109 and 40 mmHg respectively. The presence of cardiac rhythm abnormalities may not be ruled out, however the owner declined any further investigation. The regular sinus rhythm observed during the CT scan and the normal intraoperative and postoperative ECG monitoring did not reveal abnormalities other than those observed during surgery.

To prevent or minimize the occurrence of the TCR during surgery in human medicine, the use of loco-regional anaesthesia has been proposed [1]. Because the area of the upper lips is innervated by the infraorbital nerve, the infraorbital block through the infraorbital channel approach could have been used [16].

In conclusion the occurrence of a maxillomandibulocardiac reflex (a subtype of TCR) although rare may be a potential life-threatening complication of procedures involving the sensory areas innervated by the maxillary and mandibular branches of the trigeminal nerve and it may cause bradycardia with hypotension. As in human medicine, parasympatheticolytic agents like atropine may not reverse the cardiovascular abnormalities, as these are due not only to an over activation of the vagus but also to a sympathetic depression. In case of refractory response to the conventional treatment, the use of a β1 adrenoceptor agonist may be indicated.

## Abbreviations

AV: atrioventricular
CT: computed tomography
ECG: electrocardiogram
HR: heart rate
IV: intravenous
TCR: trigeminocardiac reflex
